# Human Organotypic Lung Tumor Models: Suitable For Preclinical ^18^F-FDG PET-Imaging

**DOI:** 10.1371/journal.pone.0160282

**Published:** 2016-08-08

**Authors:** David Fecher, Elisabeth Hofmann, Andreas Buck, Ralph Bundschuh, Sarah Nietzer, Gudrun Dandekar, Thorsten Walles, Heike Walles, Katharina Lückerath, Maria Steinke

**Affiliations:** 1 Department of Tissue Engineering and Regenerative Medicine, University Hospital Wuerzburg, Wuerzburg, Germany; 2 Translational Center ´Regenerative Therapies for Oncology and Musculoskeletal Diseases`Wuerzburg, branch of the Fraunhofer Institute Interfacial Engineering and Biotechnology (IGB), Wuerzburg, Germany; 3 Department of Nuclear Medicine, University Hospital Wuerzburg, Wuerzburg, Germany; 4 Department of Cardiothoracic Surgery, University Hospital Wuerzburg, Wuerzburg, Germany; 5 Department of Nuclear Medicine, University Hospital Bonn, Bonn, Germany; Technische Universitat Dresden, GERMANY

## Abstract

Development of predictable *in vitro* tumor models is a challenging task due to the enormous complexity of tumors *in vivo*. The closer the resemblance of these models to human tumor characteristics, the more suitable they are for drug-development and –testing. In the present study, we generated a complex 3D lung tumor test system based on acellular rat lungs. A decellularization protocol was established preserving the architecture, important ECM components and the basement membrane of the lung. Human lung tumor cells cultured on the scaffold formed cluster and exhibited an up-regulation of the carcinoma-associated marker mucin1 as well as a reduced proliferation rate compared to respective 2D culture. Additionally, employing functional imaging with 2-deoxy-2-[^18^F]fluoro-D-glucose positron emission tomography (FDG-PET) these tumor cell cluster could be detected and tracked over time. This approach allowed monitoring of a targeted tyrosine kinase inhibitor treatment in the *in vitro* lung tumor model non-destructively. Surprisingly, FDG-PET assessment of single tumor cell cluster on the same scaffold exhibited differences in their response to therapy, indicating heterogeneity in the lung tumor model. In conclusion, our complex lung tumor test system features important characteristics of tumors and its microenvironment and allows monitoring of tumor growth and -metabolism in combination with functional imaging. In longitudinal studies, new therapeutic approaches and their long-term effects can be evaluated to adapt treatment regimes in future.

## Introduction

Lung cancer is the major cause of cancer related death worldwide [1]. Despite vigorous research efforts, the 5 year-survival rate of lung cancer patients remains at about 15% [2]. In recent years improved molecular characterization of non-small cell lung cancer (NSCLC) led to a better understanding of certain driver mutations and new targeted treatment options. The success of anti-tumor therapy with epidermal growth factor receptor (EGFR) inhibitors for patients harboring activating EGFR mutations is only one example emphasizing the potential for personalized treatment approaches [3]. Nevertheless, only the small subset of NSCLC patients carrying this genetic mutation benefits from such treatments and secondary mutations often lead to drug resistance, followed by tumor progression and ultimately death of the patients [4]. Thus, there is an urgent scientific and medical need for a refined understanding of lung tumor biology including mechanisms of tumor heterogeneity and resistance to provide optimal strategies and promising drug combinations [5] to treat lung cancer in an individual patient.

Despite extensive preclinical testing, many newly developed oncologic drugs fail clinically—predominantly at a late stage in phase II and III trials—making drug-development a cumbersome and extremely cost- and time-consuming process. This is especially prominent in the field of oncology [6–10]. One reason for this is the lack of appropriate predictive model systems to distinguish efficient from non-efficient drug compounds early and cost-effectively [11–13]. The response of tumors to drugs is influenced by a complex interaction of several factors, including tissue-specific microenvironment, cell type specific response and mechanical stimuli [14–17]. All of these are poorly reflected by conventional two-dimensional (2D) cell cultures. Animal models better reproduce *in vivo* tissue characteristics and have massively contributed to our understanding of tumor biology. However, the transferability of results into the human setting is limited owing to e.g. the chimeric nature of the resulting tumors [18–22]. Thus, there is still an urgent need for reliable, organotypic tumor models with high predictive power regarding clinical effectiveness and with the potential to use primary patient-derived cells for testing of individual responses [13, 23].

Three dimensional (3D) human tumor models represent a promising option to bridge the gap between 2D cultures and animal models. Tissue engineering strategies offer powerful tools to build up artificial tissues and test systems. Especially, the use of tissue-specific extracellular matrix (ECM) holds great potential, as the tissue-specific composition directly influences cellular processes and identity [24, 25]. Various approaches have shown success, ranging from a small-scale alveolar-capillary barrier model on a micro-chip device [26] to hydrogel-based models [27] and *in vitro* generated transplantable rodent lung tissue from a decellularized organ scaffold [28]. So far, a focus has been on hydrogels, predominantly on Matrigel^™^, a hydrogel based on an extract from Engelbreth-Holm-Swarm mouse sarcomas [29–31], as their chemical and structural properties, such as porosity, pore size, permeability and mechanical stability, can be controlled [32, 33]. Matrigel^™^ contains basement membrane components such as laminin and collagen as well as a variety of growth factors [34], but also displays a high variability in the composition between different batches impeding reproducibility and complicating drug screening [35].

In this study, we aimed to establish a 3D organotypic lung tumor model in which lung tumor biology as well as new therapeutic strategies can be investigated and using a non-destructively a clinically relevant read-out modality. Based on an acellular rat lung scaffold and human NSCLC cell lines, our lung tumor test system provided distinct tumor nodules in a tissue-specific environment, which adapt an *in vivo*-like phenotype. Using functional imaging with 2-deoxy-2-[^18^F]fluoro-D-glucose positron emission tomography (FDG-PET), a widely used method for diagnosis, staging and prognostication of lung cancers [36], tumor cell clusters could be detected and tracked over time and response to treatment to with a targeted therapy could be monitored.

## Materials and Methods

### Ethics statement

All animals were bred in the in-house animal facility and received standard animal care and proper attention in compliance with the Guide for Care and Use of Laboratory Animals published by the National Institute of Health (NIH publication no. 85e23, revised 1996). No approval of the Institutional Animal Care and Use Committee (IACUC) was required for the organ removal according to the animal welfare act §4. Only the minimal number of 35 animals necessary to produce reliable scientific data was used. All animals were sacrificed by exposure to CO_2_ without any prior interventions. Of the sacrificed animals not only the lung but also further organs for other research purposes were explanted.

### Preparation of rat lungs

8 to 9 weeks old Lewis rats (Charles River, Germany) were euthanized by exposure to CO_2_ (Cp pharma, Germany). PBS containing 50 U/ml Heparin (Ratiopharm, Germany) was perfused through the right ventricle to prevent blood clotting. Lung and heart were excised en bloc. The pulmonary artery and trachea were cannulated with 18G and 14G catheters (Braun, Germany), respectively. An additional catheter was sutured into the left atrium of the heart to establish a circulation.

### Decellularization of lungs and characterization of lung scaffolds

In order to generate acellular lung scaffolds, we tested four different protocols (detailed below) and finally chose the H_2_O—sodium deoxycholate (SDC) procedure for further studies, which is a protocol adapted from the generation of the porcine BioVaSc^®^-TERM [37] depicted in S1 Fig. The decellularization process was performed under non-sterile conditions at room temperature. Peristaltic pumps (Ismatec, Germany) were used for the vascular perfusion via the pulmonary artery and were controlled by a pressure sensor. The flow rate was automatically adjusted to maintain a pressure according to the specific protocol. The resulting scaffolds were exposed to 25 kGy gamma-radiation for sterilization performed by the company BBF Sterilisationsservice GmbH (Rommelshausen, Germany).

#### H_2_O –SDC—Protocol

Isolated lungs were instilled with 3 mL deionized water and perfused with 500 mL deionized water at a mean pressure of 15 mmHg. After each 100 mL of fluid the perfusion was stopped and 3 mL fresh deionized water was infused via the trachea into the lung. This perfusion process was repeated until the vasculature had been rinsed with 500 mL deionized water. Lungs were then filled with 3 mL of deionized water and incubated in deionized water at 4°C overnight on a rocking platform shaker. The next day, 3 mL 2% SDC/H_2_O (deionized) were applied to the trachea; subsequently, lungs were perfused with 2% SDC/H_2_O at a pressure of 20 mmHg. After 100 mL decellularization fluid had run through the vasculature, perfusion was interrupted; lungs were filled with 3 mL 2% SDC and allowed to deflate before continuing the process of perfusion until 500 mL 2% SDC had been perfused through the lungs. Following immersion and incubation in 2% SDC/H_2_O at 4°C on shaker overnight, lungs were washed by perfusion of 500 mL PBS at a mean pressure of 20 mmHg. Every 100 mL perfused, lungs were filled with 3 mL PBS via the trachea. To remove residual genomic DNA from the matrix, 3 mL PBS with calcium and magnesium ions containing 333.33 μg/mL DNaseI were injected into the trachea, pulmonary artery and the lungs were incubated in this solution at 4°C overnight on a rocking platform shaker after which it was stored in PBS.

#### SDS-Protocol [28]

Lungs were perfused via the pulmonary artery maintaining a mean pressure of 30 mmHg. Lungs were successively perfused with 0.1% SDS in deionized water for 2 h, deionized water for 15 min and 1% Triton X-100 in deionized water for 10 min. Next, the vasculature was rinsed with PBS for 60 min. Finally, lungs were stored in PBS containing 1% Penicillin/Streptomycin at 4°C.

#### CHAPS-Protocol [38]

For decellularization, 3 mL PBS containing 8 mM 3-[(3-Cholamidopropyl) dimethylammonio] -1-Propanesulfonate (CHAPS), 1 M NaCl and 25 mM EDTA were instilled into the trachea after which the vasculature was perfused with PBS for 30 min. Subsequently, 500 mL of the CHAPS containing decellularization solution were perfused through the vasculature; lungs were then rinsed with 500 mL PBS. A mean pressure of 20 mmHg was maintained for all perfusion periods. Following, lungs were incubated in PBS containing 1% Penicillin/ Streptomycin at 4°C for 48 h, changing the solution every 24 h, under constant agitation. Next, a DNase digestion step was performed over night as described above at 4°C under constant agitation, after which the scaffolds were stored in PBS.

#### Triton—SDC—Protocol [39]

Resected lungs were incubated in deionized water for 1 h at 4°C. Following, 3 mL deionized water were manually injected into the vasculature via the pulmonary artery and subsequently into the trachea using a 5 mL syringe. After deflation, this process was repeated 4 further times. Next, 3 mL 0.1% Triton X-100/ H_2_O (deionized) were instilled into both the trachea and the vasculature, the lungs were submerged in this solution and incubated for 24 h at 4°C under constant agitation. The next day, lungs were washed five times with 3 mL deionized water as before. Thereafter, 3 mL 2% SDC were injected into both, trachea and pulmonary artery, and the organ was immersed and incubated in this solution for 24 h at 4°C. Following another washing step (5-times 3 mL deionized water), lungs and vasculature were filled and submerged with 3 mL 1 M NaCl in deionized water containing 5% Penicillin/ Streptomycin for 1 h at RT under constant shaking and washed again (5 times 3 mL deionized water in both trachea and pulmonary artery). The scaffold was incubated in PBS containing calcium and magnesium ions and 333.33 μg/mL DNaseI at 4°C overnight, removed from this solution the next day and stored in PBS.

For the analysis of lung scaffolds, acellular matrices as well as native lungs were sectioned as illustrated in S2 Fig. and used for quantification of DNA-, collagen-, and elastin-concentration as well as for histology and ultrastructure examination as annotated in S2 Table.

To detect residual DNA in acellular lung matrices, the dsDNA PicoGreen Assay (Life Technologies, Germany) was performed according to the manufacturer’s instructions. Collagen and elastin content was assessed using the Bicolor Collagen Assay and Fastin Elastin Assay (Biocolor, UK), respectively, according to the manual. The percentage of airspace area in Hematoxylin-Eosin (HE) stained lung slices was calculated using Image J [40].

### Histology,immunohistochemistry and immunofluorescence

Decellularized and recellularized lungs were fixed with 4% paraformaldehyde (PFA) at 4°C overnight, paraffin-embedded and sectioned at 5 and 7 μm thickness. HE, Elastica van Gieson, Movat’s pentachrome and Feulgen staining were performed according to standard protocols.

For immunohistochemistry and –fluorescence, slides were rehydrated and boiled in 10 mM sodium citrate buffer (pH = 6.0) for 20 min to retrieve antigens. Endogenous peroxidases were inactivated using 3% H_2_O_2_ for immunohistochemical staining using 3´- 3´-Diaminobenzidine (DAB). Primary antibody detection and chromogenic visualization with DAB was performed using the DCS Super Vision 2 HRP-Polymer-Kit (DAKO, Germany) according to manufacturer’s instructions. Stained sections were dehydrated, cleared, and cover-slipped. For immunofluorescent staining, sections were pre-incubated in 5% normal donkey serum diluted in antibody incubation buffer (DCS Innovative Diagnostik-Systeme, Germany). Primary antibody incubation was performed at 4°C overnight. The samples were washed three times in PBS containing 0.5% Tween-20 followed by secondary antibody incubation for 1 h at room temperature, washed again three times and cover slipped in a mounting medium containing 0.1% DAPI. To control the specificity of primary antibodies, negative controls (omission of primary antibodies) were performed for each experiment. All antibodies and dilutions used are listed in S1 Table. Photographs were taken using the BZ-9000 BIOREVO System (Keyence, Germany) and a confocal laser scanning microscope (SP8, Leica, Germany).

### Recellularization of lung scaffolds

Tumor models were generated by injection of 10, 15 and 25 x 10^6^ A549 or HCC827 cells resuspended in 1.5 ml RPMI containing 10% or 20% FCS, respectively, into the trachea of lung scaffolds. Lungs were incubated for 2 h in medium at 37°C and 5% CO_2_ and then transferred into a glass bottle and cultured in 80 ml medium. Medium was exchanged every second day during a culture period of 14 days.

### ^18^F-FDG uptake (2D cell cultures)

200,000 HCC827 or A549 cells were incubated with 1x10^6^ counts per minute (cpm) of ^18^F-FDG for 60 min. After incubation on ice to stop uptake and washing with PBS twice, remaining intracellular ^18^F-FDG activity was quantified using a semi-automated gamma-counter (Wallac 1480-Wizard, Perkin Elmer, Germany). Decay- and background corrected data were expressed as percent of initially added activity.

### ^18^F-FDG PET imaging of lung tumor models

Tumor models were cultured for 11 days and then treated with 1 μM gefitinib (AstraZeneca, Germany) for 72 h. For FDG-PET, each model was incubated in 25 MBq ^18^F-FDG/ 30 ml PBS for 1 h at 37°C under slight agitation, washed three times with PBS for 5 min and transferred in a sterile Petri dish for imaging. Data were acquired for 15 min using a dedicated small-animal PET-scanner (Inveon; Siemens Preclinical Solutions, Germany) and reconstructed using ordered subset expectation maximization 2D (OSEM 2D) algorithm. Tumor-to-background ratios (TBR) were determined by drawing 3D regions of interest (ROI) around individual tumor nodule or healthy tissue (background) using “a Medical Image Data Analysis Tool” (AMIDE)-software (http://amide.sourceforge.net/). Tumor models were imaged at 11 days of culture (“baseline”), 24 h (“24 h”) and 72 h (“72 h”) after treatment initiation (n = 3 for each cell line) by ^18^F-FDG-PET scanning for 15 min.

### Statistical analysis

Statistical analysis was performed using the non-parametric tests Man Whitney U, Kruskal-Wallis test and post-hoc Wilcoxon rank rum tests. All tests were performed using the open-source software R (CRAN, The Comprehensive R Archive Network).

## Results

### Establishment of a decellularization protocol for rat lungs preserving lung architecture, basement membrane and extracellular matrix (ECM) components

In order to determine the most suitable protocol for complete decellularization of rat lungs and optimal preservation of matrix structure and components, a protocol adapted from the decellularization of porcine jejunum established by our group [37] was compared to three previously published procedures [28, 38, 39] (S1 Fig).

**Fig 1:**
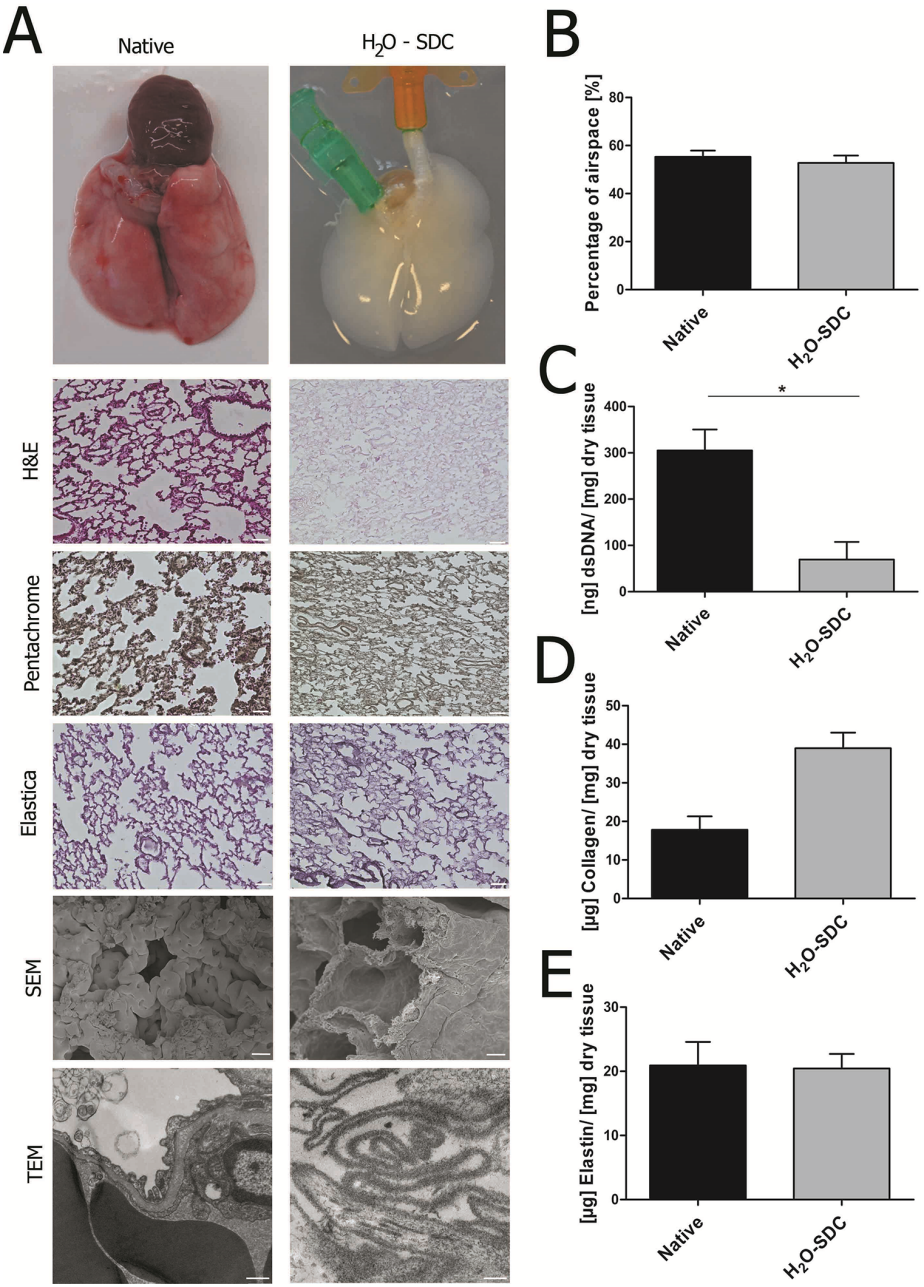
Comparison of native and decellularized rat lungs. (A) After decellularization using the H_2_O-SDC protocol, no cellular remnants were observed while the alveolar architecture and extracellular matrix proteins are well-preserved as shown in histological and ultrastructural analyses (SEM, TEM). Conservation of the alveolar-capillary basement membrane was verified using SEM and TEM. (B) Quantification of the percentage of airspace revealed no difference between decellularized and native lungs. (C) DNA content was significantly reduced after decellularization (p = 0.04, Wilcoxon rank sum test). (D-E) Higher collagen concentration was found in the acellular scaffold, while elastin was maintained at similar levels to native lung tissue. Scale bars in histological images: 50 μm; scale bars in SEM images: 10 μm, scale bars in TEM images: 500 nm; data in B-E are presented as arithmetic means ± SEM; *p<0.05, Kruskal-Wallis test; n = 5.

Our protocol utilizing H_2_O in a first step, followed by sodium deoxycholate (H_2_O-SDC), generated a scaffold of macroscopically white appearance (Fig 1A). Successful removal of cells and maintenance of lung architecture, including the basement membrane, was verified on the light microscopic and ultra-structural level employing scanning and transmission electron microscopy (SEM, TEM) (Fig 1A). In order to quantify structural preservation, the percentage of airspace in the decellularized scaffolds was assessed using H&E staining and compared to native tissue. Approximately half of the area of cross sections of distal native rat lung tissue consisted of air (55.3 ± 2.6%) and no significant difference could be measured in acellular lungs generated by H_2_O-SDC (52.8 ± 3.0%) (Fig 1B). DNA concentration was significantly reduced after decellularization (Fig 1C, p = 0.04) in accordance with the absence of nuclei in H&E staining. Regarding ECM components, collagen and elastin are of major importance in the lung, as they ensure the stability and elastic recoil of the lung. Compared to native rat lung tissue, decellularized matrices exhibited a higher collagen concentration by tendency and comparable amounts of elastin (Fig 1D and 1E). In line with this, the presence of collagen and elastic fibers could be visualized using Movat’s pentachrome, Elastica van Gieson and immunohistochemical staining (S4 and S5 Figs). Comparing the H_2_O-SDC protocol to the lung decellularization protocols published previously, only the protocol of Price et al. (Triton-SDC) [39] displayed a similar degree of ECM preservation. However, this decellularization method resulted in a higher variation in all analyses compared to H_2_O-SDC-treated matrices (S3 and S4 Figs).

### Tumor cell cluster on the decellularized lung scaffold exhibit *in vivo*-like tumor characteristics

**Fig 2:**
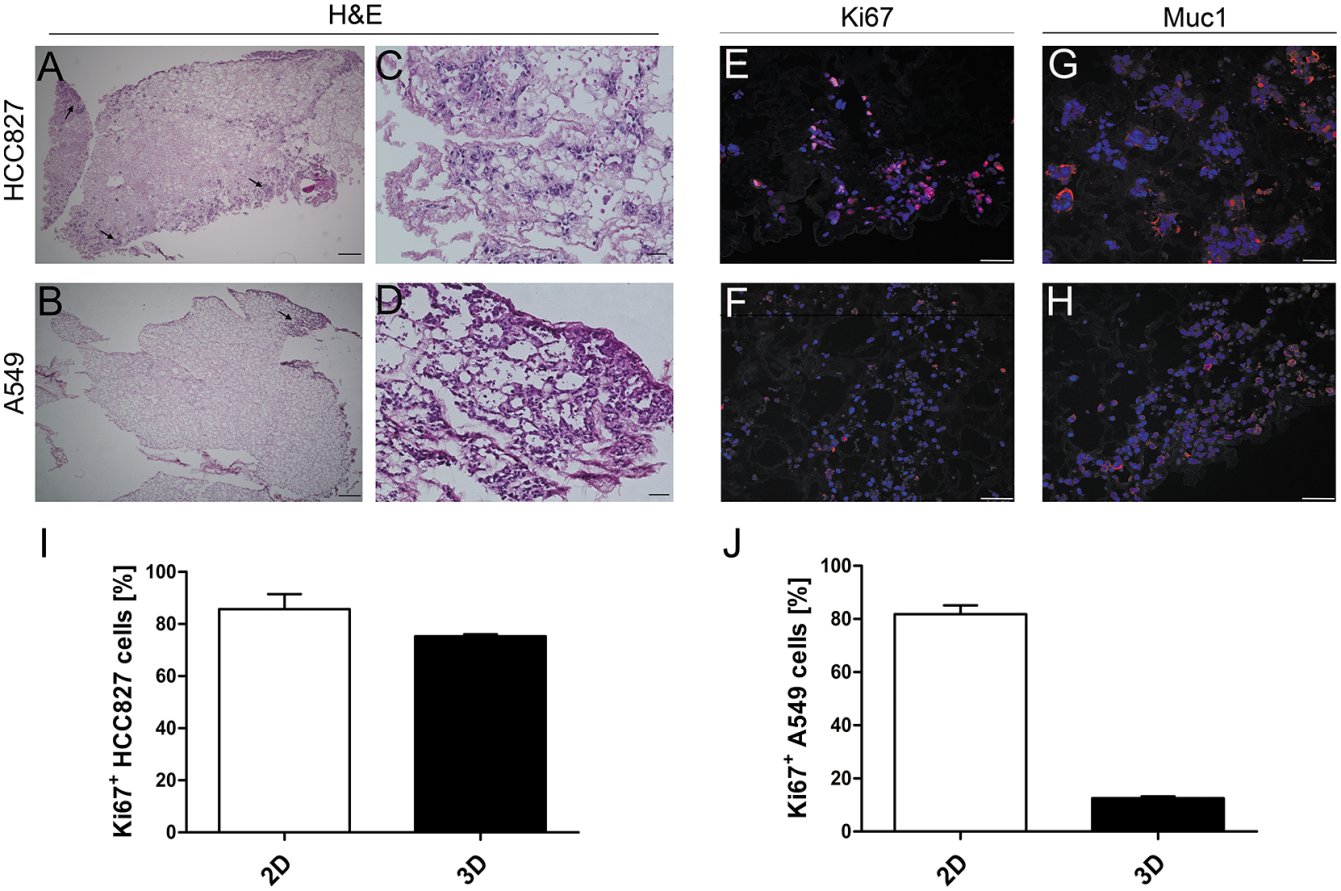
Tumor cell lines form nodules with characteristics of *in vivo* tumors on the lung scaffold. HCC827 and A549 cells were introduced through the trachea for recellularization of the airway structures. Both cell lines were able to repopulate the acellular lung scaffold. After 14 days of static culture, HCC827 (A, C) cells formed dense “tumor-like” clusters at the distal periphery of the lung. In contrast, A549 cells (B, D) displayed a scattered colonization, with more tumor-like appearance and higher tendency of cell accumulation at the periphery. Tumor cells of both cell lines were still proliferative after 14 days in static culture (E, F) and showed a high expression of Mucin-1 (G, H). Scale bars: 50 μm. (I, J) Proliferation of tumor cells was quantified by the number of Ki67-positive cells. HCC827 cell grown on the 3D scaffold exhibited a slightly reduced percentage of proliferative cells compared to 2D cell culture (75.25 ± 0.74% vs. 85.7 ± 0.06% p = 0.057, Wilcoxon rank sum test) (I), while only a low percentage of A549 cells was proliferative in the 3D scaffold (12.52 ± 0.91% vs. 81.78 ± 0.03, p = 0.057, Wilcoxon rank sum test) (J). Data are presented as arithmetic means ± SEM; n = 3.

For repopulation of decellularized scaffolds two established human NSCLC cell lines were chosen because they provide foundation for standardization of such a system and were approved in other studies to be relevant representatives of the clinically found tumors [41]. While the A549 cells harbor wildtype EGFR, a mutant KRAS and show a very weakly differentiated phenotype, HCC827 cells possess an activating EGFR mutation, wildtype KRAS and represent a more differentiated state [42]. Upon static culture for 14 days tumor cells repopulated the matrix in-homogenously. While HCC827 cells displayed a more cluster-like pattern (Fig 2A), a scattered distribution of single cells was predominant in A549 cells (Fig 2B). Additionally, both cell lines formed several tumor clusters with “nodule-like” appearance predominantly at the periphery (Fig 2C and 2D). Proliferating Ki67-positive tumor cells were observed throughout the whole scaffold (Fig 2E and 2F). Compared to the respective 2D cell culture, the proliferation rate of tumor cell lines grown on the 3D lung scaffold was decreased, in HCC827 cells to 75.3% *vs*. 85.7% (p = 0.057) and in A549 cells to 12.5% *vs*. 81.8% (p = 0.057) (Fig 2I and 2J). This complies with a low number of proliferating cells in adenocarcinoma *in vivo*. Moreover, the expression of mucin1 (Muc1), a lung carcinoma associated protein, was markedly up regulated upon 3D lung scaffold culture and displayed a depolarized expression pattern (Fig 2G and 2H) that is similar to the situation *in vivo*.

### Uptake of FDG by lung cancer cell lines in conventional 2D culture

**Fig 3:**
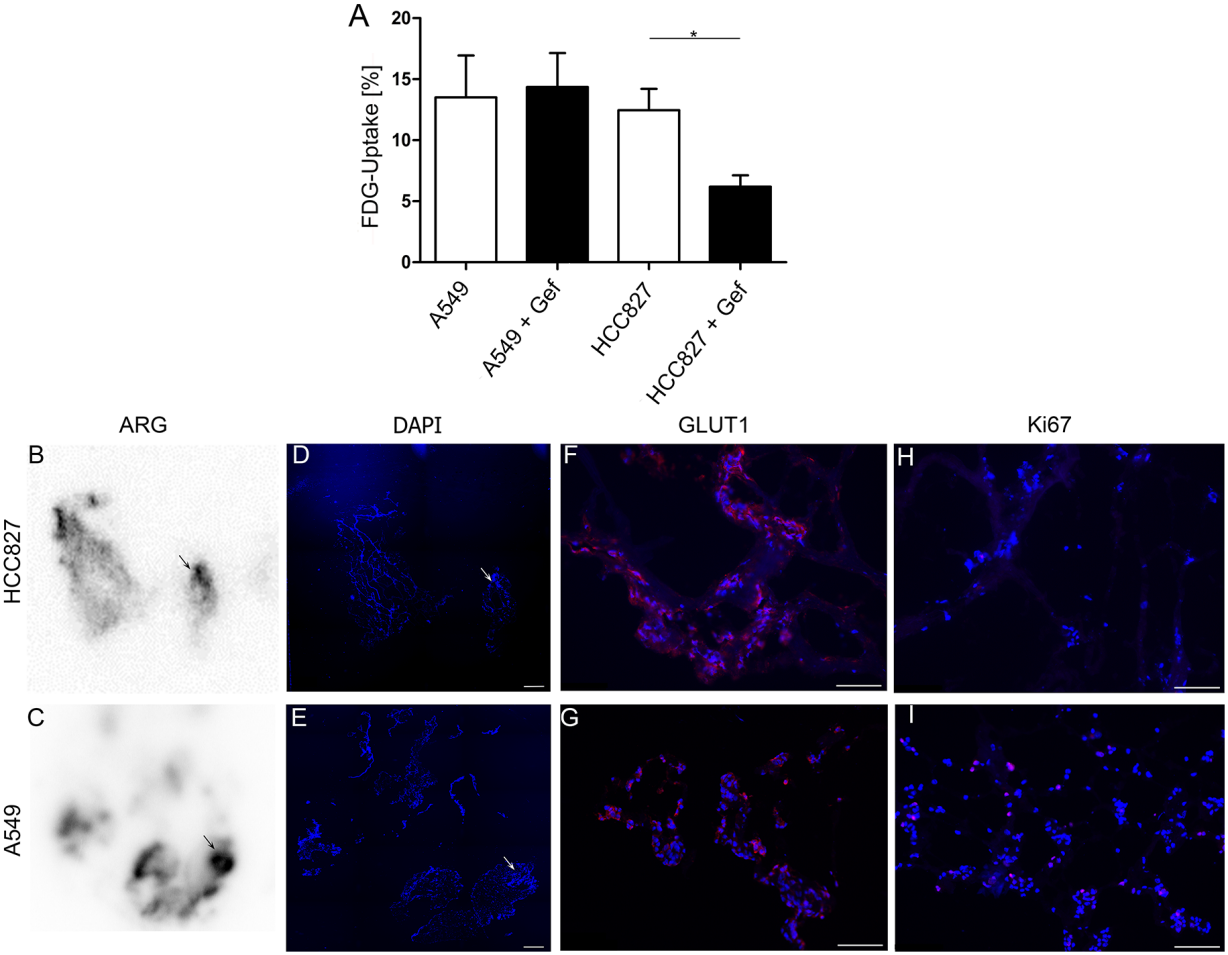
Detection of tumor nodules with FDG-PET. (A) Gefitinib-treated and untreated A549 and HCC827 cells grown in 2D were incubated with ^18^F-FDG for 60 minutes and FDG uptake was quantified using a gamma-counter. Data were corrected for background and decay and related to the initially added activity (n = 4, p = 0.02, Man Whitney U test). (B-I) Cross sections of lungs recellularized with tumor cells and incubated with ^18^F-FDG were investigated by autoradiography (ARG). Regions of high radioactive intensity correlated with presence of tumor cells (arrows in B, C, D, E). Cells of both cell lines strongly expressed GLUT1 (F, G) while only a low amount of the tumor cells was proliferative as shown by Ki67 staining (H, I). Data are presented as arithmetic means ± SEM; *p<0.05, Man Whitney U test; scale bars: 50 μm. One representative experiment out of 3 is shown.

In order to establish a non-destructive tool to monitor tumor metabolism and drug responses in the 3D tumor model, we first tested the feasibility of using FDG retention as read out in 2D cell cultures. Both, HCC827 and A549 cells retained considerable and similar amounts of FDG in 2D cell culture, reaching 12.5% and 13.5%, respectively, after 60 min (Fig 3A). To assess the suitability of FDG to monitor treatment responses, uptake of cells treated with the EGFR-inhibitor gefitinib was compared to that of untreated ones. While gefitinib had no impact on FDG uptake in EGFR-wildtype A549 cells (13.5 ± 2.4% *vs*. 14.4 ± 2.8%), it significantly reduced FDG retention by 50% (12.5 ±1.2% *vs*. 6.2 ± 0.9%, p<0.02) in EGFR-mutant HCC827 after 60 min of incubation (Fig 3A).

### FDG-PET is suitable to detect tumor nodules in a 3D lung tumor model

**Fig 4:**
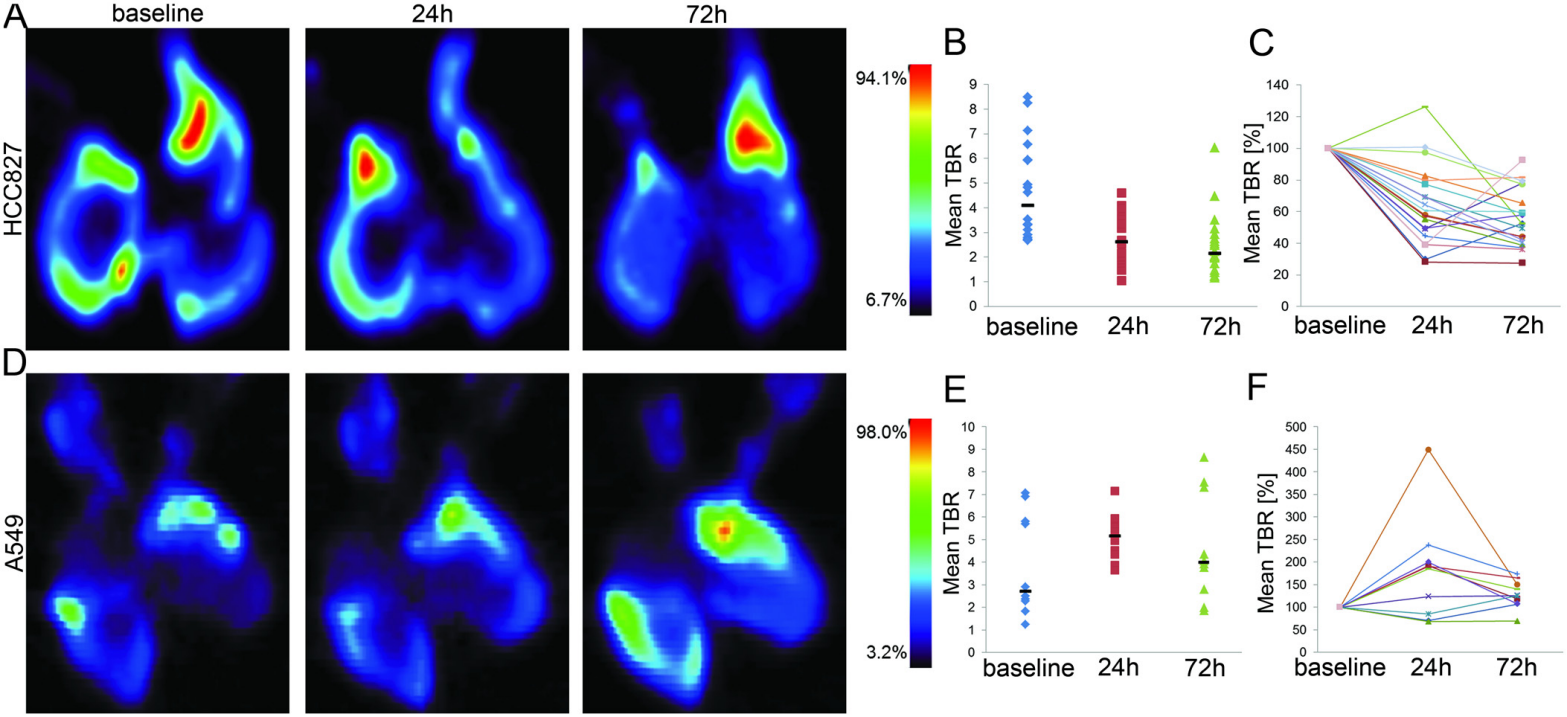
Monitoring of treatment response to targeted therapy with FDG-PET in lung tumor models. Lungs recellularized with HCC827 (top panel, A) or A549 (bottom panel, D) were incubated with ^18^F-FDG-PET for 60 min and imaged using a μPET-scanner. Coronal views of exemplary lung scaffold cultures (n = 3) at baseline and following 24 h or 72 h treatment with gefitinib are shown. 3D regions of interest were defined at individual tumor nodules or scaffold tissue and mean tumor-to-background ratios (TBR) were calculated (B, E). Horizontal bars depict median values for the mean TBRs of individual nodules at baseline, 24 h and 72 h. (C, F) Relative change of tracer uptake in individual tumor clusters compared to ^18^F-FDG-PET-intensity at baseline is shown. Intensity of tracer uptake decreased over the course of 72 h treatment with gefitinib in HCC827-, while no dramatic effect was detected in A549-seeded lungs.

Based on this, we investigated if FDG uptake of tumor cell clusters on the 3D lung scaffold could specifically be detected using PET imaging. As proof of principle, autoradiography of cross sections of repopulated lungs revealed distinct regions of high radioactivity in the periphery (Fig 3B and 3C). Subsequent staining with DAPI confirmed the presence of large tumor cell clusters in these areas (Fig 3D and 3E). In accordance with this, both cell lines strongly expressed GLUT1, the glucose transporter responsible for FDG uptake (Fig 3F and 3G). In a next step, FDG-PET imaging of repopulated lungs revealed that tumor nodules could clearly be detected and distinguished from surrounding tissue (Fig 4A and 4D) with median tumor-to-background ratios (TBR) of 4.08 (range 1.9 to 8.5) for HCC827 and 2.70 (range 1.2 to 7.1) for A549, respectively (Fig 4B and 4E “baseline”).

### FDG-PET can be used for non-destructive monitoring of response to treatment

In order to assess the suitability of functional PET-imaging as a non-destructive and clinically relevant read-out in the lung tumor test system, scaffolds repopulated with either HCC827 or A549 cells were treated with 1 μM gefitinib and examined by FDG-PET at baseline, 24 h and 72 h post treatment induction. Compared to the pre-therapeutic uptake, median TBR of HCC827 tumor nodules was reduced by 36% (median TBR 2.6, range 1.0 to 4.6) 24 h and by 48% (median TBR 2.13, range 1.2 to 4.5) 72 h after treatment induction (Fig 4A and 4B). In contrast, median FDG uptake by A549 nodules increased from 2.70 at baseline to 5.15 (range 3.7 to 7.2; 191%; 24 h) and 3.98 (range 1.9 to 8.6; 147%; 72 h) in the presence of gefitinib (Fig 4D and 4E). In line with this, only very few proliferative HCC827 cells could be detected after 72 h of gefitinib treatment, while the proliferation of A549 cells was not affected (Fig 3H and 3I).

Tracking individual HCC827 nodules, already 24 h after treatment induction a significant reduction in FDG retention was observed in all cases but one; further 48 h of treatment (72 h time point) resulted in an additional moderate decrease in most (15/20) nodules and in enhanced tracer retention in five cases. However, FDG uptake by these five nodules still was significantly lower compared to baseline (Fig 4C). For tumors formed by A549 cells severely enhanced FDG uptake was observed in 7/10 cases after 24 h and the remaining three only showed a moderate reduction (15–30%). Further exposure to gefitinib led to a slight drop in tracer intensity in 6/10 and a slight increase or no change in 4/10 cases; FDG uptake was significantly higher after 72 h of treatment in 9/10 nodules compared to baseline (Fig 4F). In conclusion, the targeted treatment approach using gefitinib reduced FDG uptake and retention specifically in the sensitive HCC827 nodules while the resistant A549 tumor models showed increasing FDG uptake during the 72 h treatment period. Moreover, FDG-PET imaging revealed that individual tumor nodules varied in their drug response.

## Discussion

In this study, we introduce an organotypic 3D human lung cancer model that can be used to explore basic tumor biology and metabolism and to simulate a biomarker-guided therapy. To our knowledge, this is the first demonstration that a thorough investigation of an *in vitro* tumor model is feasible using non-destructive molecular PET-imaging. We are convinced that this innovative combination of advanced tissue engineering with functional imaging will enable longitudinal studies regarding tumor growth, -metabolism and –heterogeneity and the evolution of resistance as well as the evaluation of new therapeutic strategies and long-term effects of treatment.

For the establishment of cancer models with high predictive power, the generation of an organotypic microenvironment, in which the tissue-specific architecture, including tissue perfusion, and the ECM are reproduced is critical [43–47]: while the ECM constitutes the anchorage for cells and is able to influence cellular phenotypes [25, 48], the basement membrane might influence tumor cell behavior [49] and crossing this barrier is an important step in tumor progression and metastasis [50, 51]. Together, an organotypic environment has an impact on cell morphology, growth, differentiation, drug response, signaling and the malignant phenotype of tumor cells.

One promising approach to recreate tissues *in vitro* is the decellularization of donor organs and repopulation of these cell-free scaffolds with human cells [37, 43, 52–55]. Comparing four decellularization protocols for rodent lungs [28, 38, 39], we found that tracheal and vascular application of H_2_O, followed by SDC was superior in terms of preservation of structure and ECM components. Additionally, standardized generation of organotypic scaffolds and test systems with our H_2_O-SDC protocol seemed feasible. This low variation might be due to the application of a distinct perfusion pressure instead of manual injection, which allowed the use of an automated pressure-controlled applicator. Upon repopulation of scaffolds, lung tumor cell lines formed distinct tumor nodules predominantly at the periphery even under simplified static culture conditions. This location corresponds to the origin of adenocarcinomas *in vivo* [56, 57]. Additionally, tumor cells in our model exhibited specific characteristics of complex tumor tissues, such as a reduced proliferation rate (compared to the respective 2D cultures) and an upregulated and depolarized expression of the carcinoma-associated marker MUC1 which is associated with dedifferentiation and oncogenic signaling [58]. The extremely high proliferation rate of conventional 2D models contrasts with that of most tumors in patients and is a major challenge in drug testing [59–61]. Our observations are in agreement with a published study in which comparison of 3D gene expression data sets of A549 cells in Matrigel^™^-based models and on an acellular rat lung under perfusion revealed a clear benefit over 2D cell cultures: The gene expression pattern of cells on the lung scaffold showed high similarity to tumors of patients with poor prognosis while the Matrigel^™^-based model resembled the phenotype of patients with better prognosis [62]. Thus, our organotypic lung tumor model seems to be a suitable system for the reliable in depth analysis of tumor biology and the testing of drugs and treatment strategies. A clear advantage over Matrigel^™^ tumor models is based on the reflection of the complex lung architecture, which hydrogels cannot offer. The high batch-to-batch variability and the undefined growth factor composition further complicate the standardization and validity of the gel-based tumor models [35], rendering them more suitable for less complex applications such as invasion assays in lung tumor models [63, 64]. Additionally, our organotypic model allows perfusion of the scaffold similar to the blood circulation in the physiologic situation [28, 38].

In order to further validate our model and establish a non-destructive, clinically relevant read-out that allows long-term studies, we aimed to set up functional imaging with FDG-PET for the lung tumor models. Proof of concept experiments in 2D cultures demonstrated specific FDG uptake by NSCLC cell lines which reduced FDG retention upon treatment in EGFR-mutant and therefore gefitinib-sensitive HCC827 cells (54, 55, 56, 57, 32) but not in the resistant A549 cells. These data suggest the feasibility of using FDG retention to monitor tumor metabolism and response to therapy and are in agreement with the proven value of FDG-PET for diagnosis, staging and monitoring of therapeutic responses of patients with lung cancer [36, 65]. Transferring these findings to the 3D lung tumor model and actual PET-imaging is not trivial, as the size of tumor nodules has to exceed the resolution limit of 1.4 mm of the μPET-scanner in order to allow analysis of individual tumor cell clusters. However, various distinct regions with high radioactivity were detected by PET and correlation of autoradiographic analyses with DAPI staining of the same section demonstrated the presence of tumor cell clusters in these regions. Thus, FDG is specifically taken up by tumor cells and not by scaffold components, proving the validity of FDG uptake in the organotypic tumor model. Furthermore, identical positioning and placement of repopulated lung scaffolds on a Petri dish and in the PET-scanner allowed tracking the development of tumor cell clusters over time. Importantly, targeted therapy with gefitinib significantly decreased FDG uptake by most HCC827 cell clusters as early as 24 h after treatment induction, but had no effect on A549 tumor nodules. This indicates that not only specific responses to therapy can be analyzed by PET in our model, but also at a very early time point at which changes in classical tumor markers presumably are not evident yet [66]. Interestingly, different HCC827 nodules on the same scaffold varied in their ability to respond to therapy and in the duration of the response, suggesting the development of an at least modest degree of tumor heterogeneity in this model after culture of 14 days.

Taken together our data show that FDG-PET-imaging of organotypic 3D human lung cancer models is a reliable method to detect and track tumor formation. Owing to the non-destructive nature and the ability to place the lung scaffolds exactly as desired, longitudinal studies are feasible. This implies that long-term effects of therapeutic regimen and the evolution of resistance can be investigated, especially when combined with immunohistochemical, flow cytometric and molecular biological analyses. Many different mechanisms of drug resistance in EGFR-mutated lung adenocarcinoma cells have been proposed [67, 68] which could be investigated further in this model. Translating such approaches to patient derived primary lung tumors would be a great step forward regarding personalized medicine. Thus, a detailed investigation of tumor metabolism and its drug-induced changes might be possible using radiotracers, such as radioactively labelled glutamine, methionine, choline or tyrosine. Moreover, new tracers for diagnosis, staging, prognostication and theranostic approaches e.g. for endo-radiotherapy can be tested, meeting a clinical need for more specific tracers than FDG: although lung cancer is one of the few conditions for which FDG-PET is accredited, false positive results due to inflammatory lesions are a common problem and false negative results might arise from lung cancer histotypes that are not FDG-avid. Ultimately these approaches could—besides deepening our knowledge of cancer biology—lead to the identification of radiotracers that could serve as biomarkers which might help to establish more effective and less toxic individualized therapies.

## Supporting Information

S1 Fig Schematic illustration of the different decellularization protocols used in this study. Each protocol utilizes a different perfusion pressure, volume or duration, respectively. Noteworthy, the SDS- and CHAPS-protocols use only the vascular system as the route of application. Protocols employing Triton-SDC and H_2_O-SDC apply decellularization solution also via the airways.
(TIF)

S2 Fig Separated and sectioned rat lung for analysis. Decellularized and native lung tissue was processed as depicted here to allow different analyses with one scaffold. Except for ultrastructural studies, each analysis was performed with tissue pieces of each part of the lung. The specific use of the single tissue pieces is listed in S1 Table.
(TIF)

S3 Fig Evaluation of decellularization protocols. (A) The percentage of airspace in the decellularized matrices was compared to native lungs to quantify the structural preservation for different decellularization protocols. While scaffolds generated using Triton-SDC and H_2_O-SDC exhibited similar values to native tissue, the CHAPS- and SDS scaffolds showed a slightly increased percentage of airspace, indicating a loss of interstitial tissue or elasticity. (B) Remaining DNA, a quality characteristic of decellularization, was reduced in all scaffolds, except the SDS-protocol (data are presented as arithmetic means ± SEM; n = 5, *p<0.05, Kruskal-Wallis test). (C) Residual DNA in the scaffold was visualized by Feulgen staining. The staining confirms presence of DNA with high regional variance in the scaffolds produced by SDS, and the absence of DNA in all other lung matrices. (D) Alcian blue staining of the acellular lungs revealed preservation of glycosaminoglycans applying SDS- and CHAPS protocol (scale bars: 50 μm).
(TIF)

S4 Fig Evaluation of extracellular matrix (ECM) components in decellularized lung scaffolds. (A) Acellular lungs generated by SDS- and CHAPS-protocols tend to contain a lower amount of collagen per mg dry tissue than the scaffolds generated with Triton-SDC or H_2_O-SDC. While these exhibited significantly higher collagen content than native rat lungs (p = 0.03 and p = 0.02, respectively, Kruskal-Wallis test). (B) A significantly reduced elastin content was detected in the SDS- and CHAPS-treated matrices compared to native tissue (p = 0.03 and p = 0.02, respectively, Kruskal-Wallis test). H_2_O-SDC-treated scaffolds displayed significantly increased elastin content compared to SDS- and CHAPS-treated tissues (p = 0.02 and p = 0.008, respectively, Wilcoxon rank sum test). Data are presented as arithmetic means ± SEM; n = 5, *p<0.05, Kruskal-Wallis test). (C) Pentachrome staining visualizes the presence of collagen in the acellular lung matrices (yellow), which is covered by a blue staining of glycosaminoglycans in the SDS- and CHAPS-treated scaffolds. (D) Elastica staining demonstrates comparable conservation of elastic fibers in all matrices. All scale bars: 50 μm.
(TIF)

S5 Fig Histologic assessment of extracellular matrix components in the decellularized lung matrices. All scaffolds showed similar and global retention of the basement membrane components collagen IV and fibronectin. In contrast, a reduced intensity for collagen I and elastin was observed in scaffolds generated with the SDS- and CHAPS– protocols. This was in accordance with the quantitative analysis detecting a lower amount of collagen and elastin in these scaffolds compared to the Triton-SDC and H_2_O-SDC generated matrices. Scale bars represent 50 μm.
(TIF)

S1 Table Antibodies used for immunohistochemical staining and immunofluorescence. (PDF)

S2 Table Analyses performed with different tissue pieces of the lung. (PDF)
