# Supplementary figures and images for: Human Organotypic Lung Tumor Models: Suitable For Preclinical 18F-FDG PET-Imaging
Source: PLoS One. 2016 Aug 8;11(8):e0160282. doi: 10.1371/journal.pone.0160282 (PMC4976941; doi:10.1371/journal.pone.0160282)

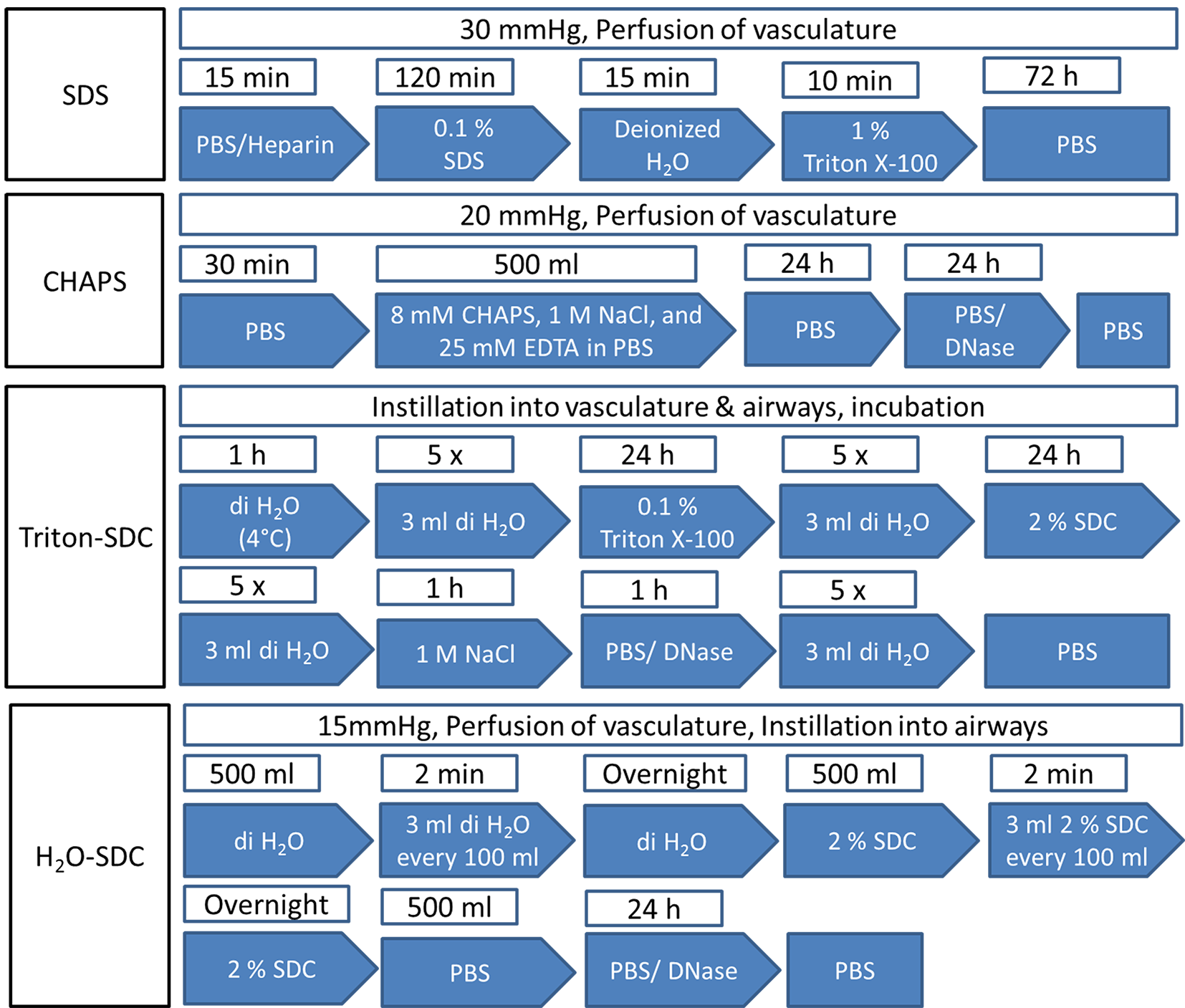

Supplement: S1 Fig — Each protocol utilizes a different perfusion pressure, volume or duration, respectively. Noteworthy, the SDS- and CHAPS-protocols use only the vascular system as the route of application. Protocols employing Triton-SDC and H2O-SDC apply decellularization solution also via the airways. (TIF) [file pone.0160282.s001.tif]

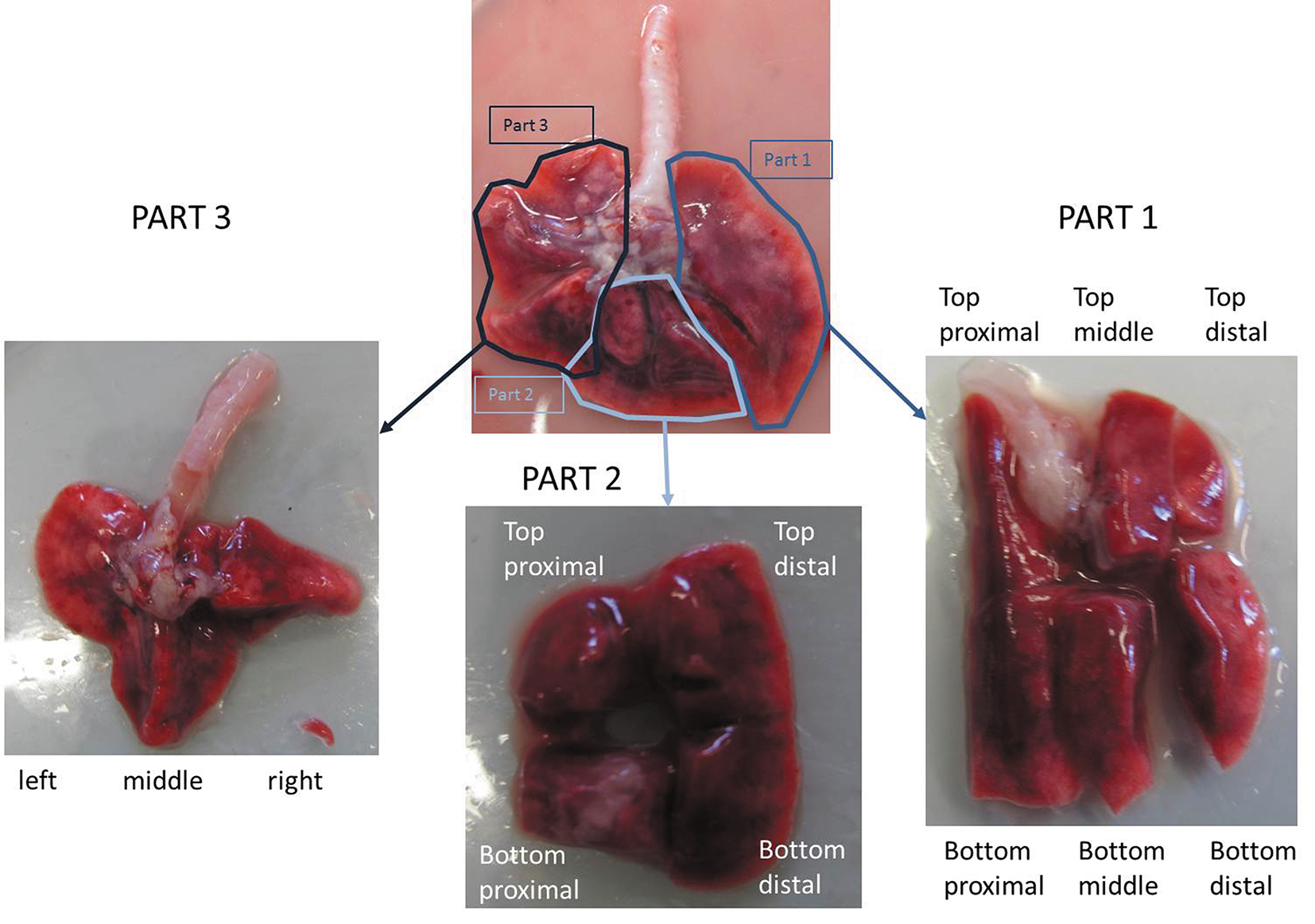

Supplement: S2 Fig — Decellularized and native lung tissue was processed as depicted here to allow different analyses with one scaffold. Except for ultrastructural studies, each analysis was performed with tissue pieces of each part of the lung. The specific use of the single tissue pieces is listed in S1 Table. (TIF) [file pone.0160282.s002.tif]

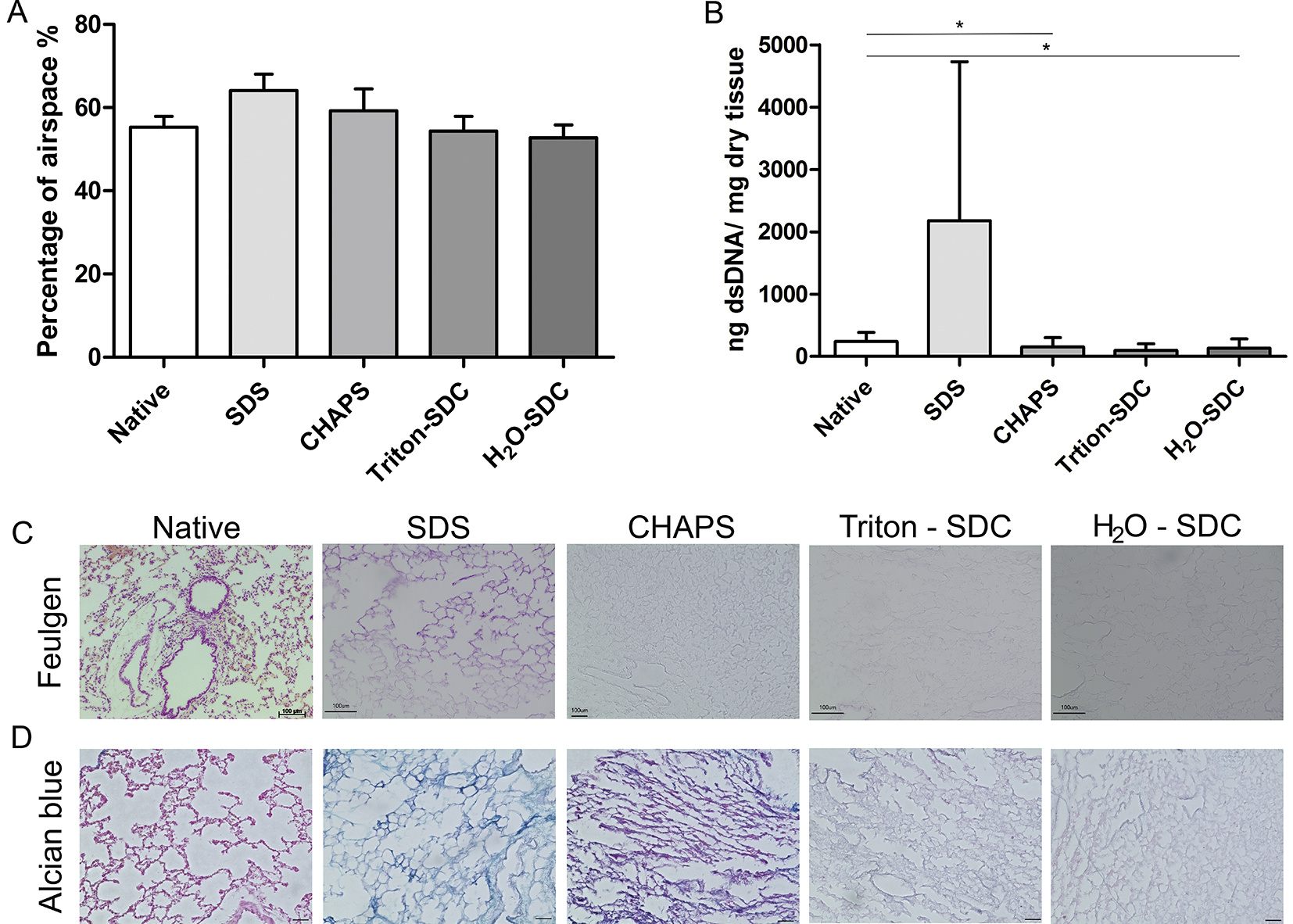

Supplement: S3 Fig — (A) The percentage of airspace in the decellularized matrices was compared to native lungs to quantify the structural preservation for different decellularization protocols. While scaffolds generated using Triton-SDC and H2O-SDC exhibited similar values to native tissue, the CHAPS- and SDS scaffolds showed a slightly increased percentage of airspace, indicating a loss of interstitial tissue or elasticity. (B) Remaining DNA, a quality characteristic of decellularization, was reduced in all scaffolds, except the SDS-protocol (data are presented as arithmetic means ± SEM; n = 5, *p<0.05, Kruskal-Wallis test). (C) Residual DNA in the scaffold was visualized by Feulgen staining. The staining confirms presence of DNA with high regional variance in the scaffolds produced by SDS, and the absence of DNA in all other lung matrices. (D) Alcian blue staining of the acellular lungs revealed preservation of glycosaminoglycans applying SDS- and CHAPS protocol (scale bars: 50 μm). (TIF) [file pone.0160282.s003.tif]

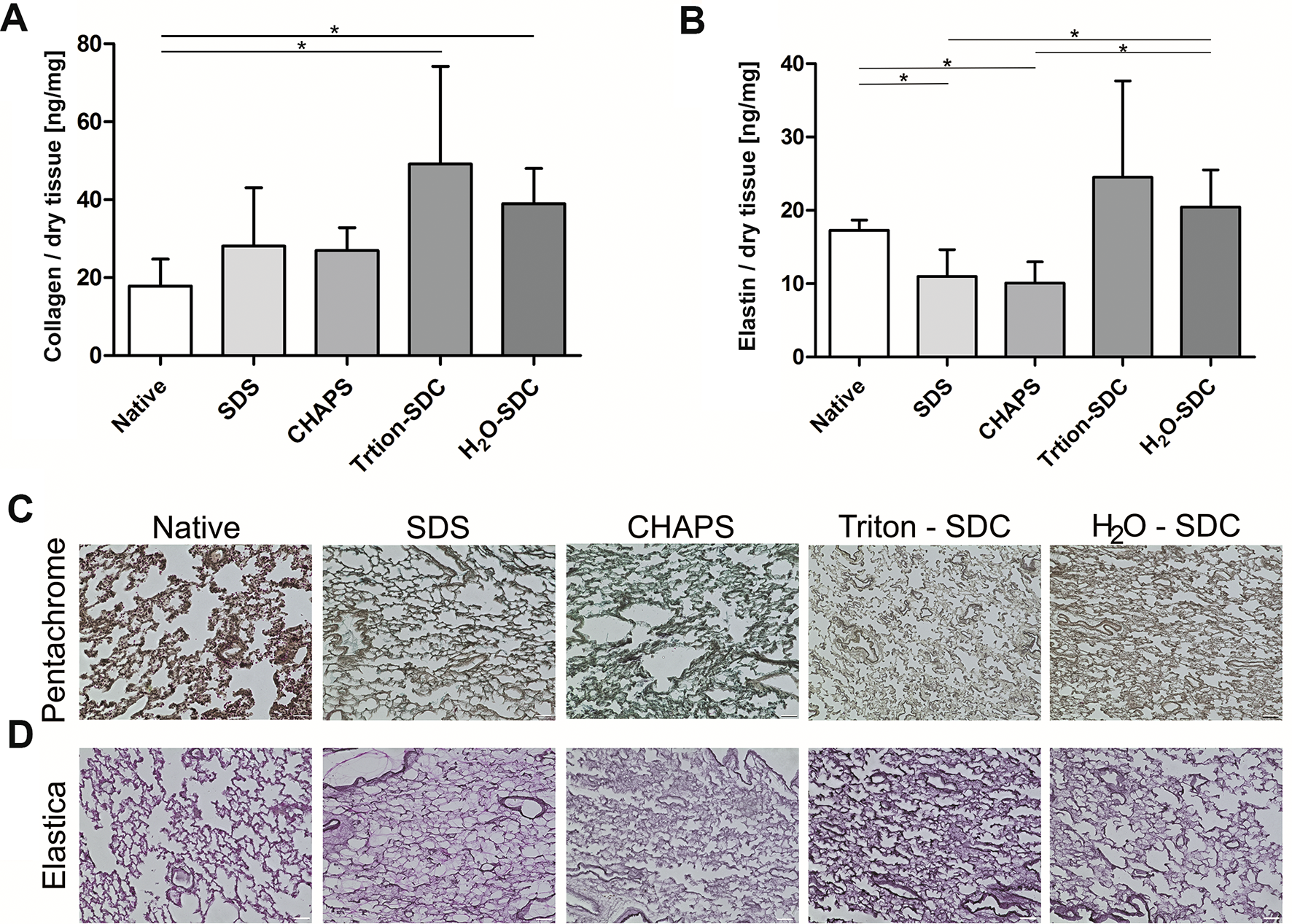

Supplement: S4 Fig — (A) Acellular lungs generated by SDS- and CHAPS-protocols tend to contain a lower amount of collagen per mg dry tissue than the scaffolds generated with Triton-SDC or H2O-SDC. While these exhibited significantly higher collagen content than native rat lungs (p = 0.03 and p = 0.02, respectively, Kruskal-Wallis test). (B) A significantly reduced elastin content was detected in the SDS- and CHAPS-treated matrices compared to native tissue (p = 0.03 and p = 0.02, respectively, Kruskal-Wallis test). H2O-SDC-treated scaffolds displayed significantly increased elastin content compared to SDS- and CHAPS-treated tissues (p = 0.02 and p = 0.008, respectively, Wilcoxon rank sum test). Data are presented as arithmetic means ± SEM; n = 5, *p<0.05, Kruskal-Wallis test). (C) Pentachrome staining visualizes the presence of collagen in the acellular lung matrices (yellow), which is covered by a blue staining of glycosaminoglycans in the SDS- and CHAPS-treated scaffolds. (D) Elastica staining demonstrates comparable conservation of elastic fibers in all matrices. All scale bars: 50 μm. (TIF) [file pone.0160282.s004.tif]

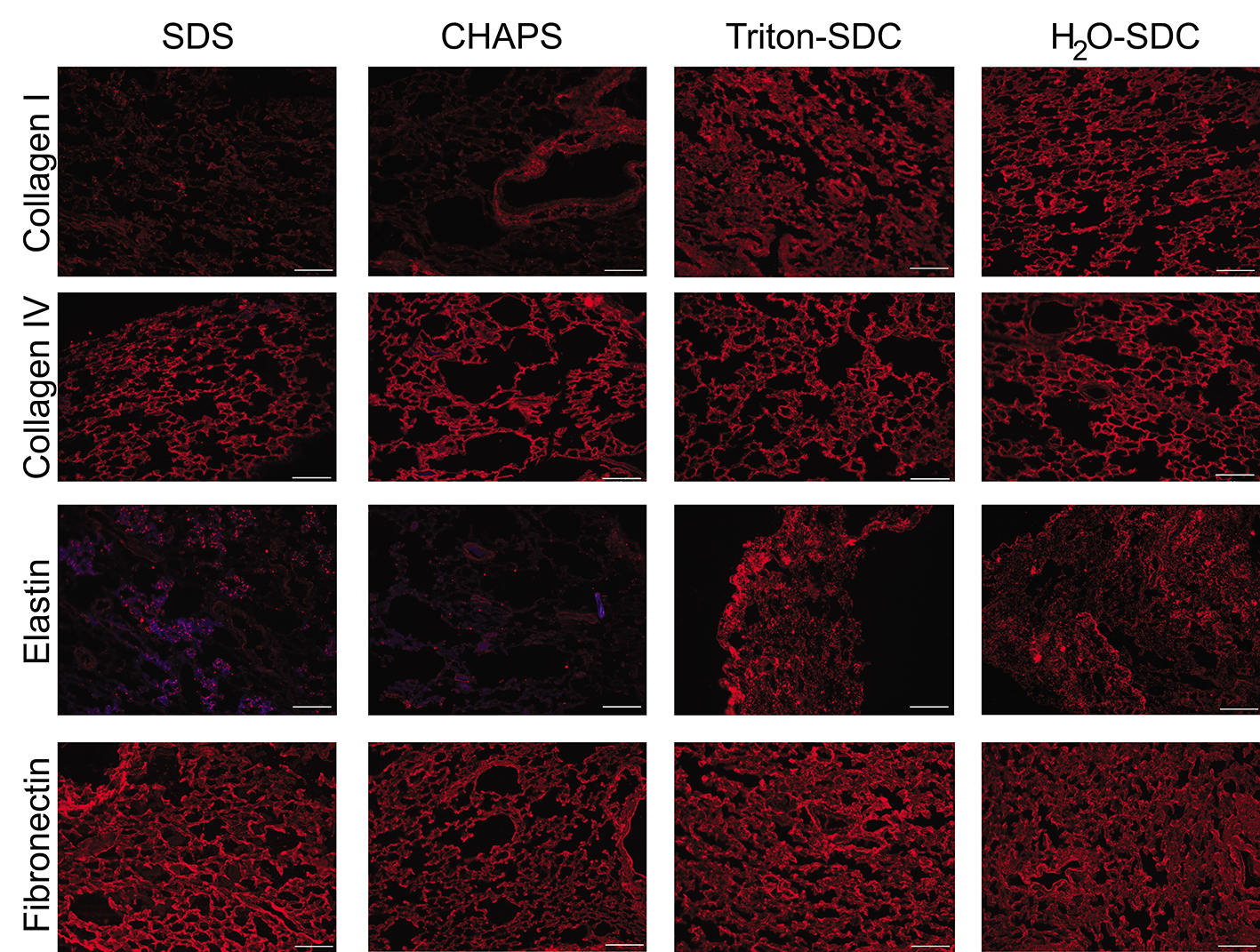

Supplement: S5 Fig — All scaffolds showed similar and global retention of the basement membrane components collagen IV and fibronectin. In contrast, a reduced intensity for collagen I and elastin was observed in scaffolds generated with the SDS- and CHAPS– protocols. This was in accordance with the quantitative analysis detecting a lower amount of collagen and elastin in these scaffolds compared to the Triton-SDC and H2O-SDC generated matrices. Scale bars represent 50 μm. (TIF) [file pone.0160282.s005.tif]
