# Supplementary material for: Human Organotypic Lung Tumor Models: Suitable For Preclinical 18F-FDG PET-Imaging
Source: PLoS One. 2016 Aug 8;11(8):e0160282. doi: 10.1371/journal.pone.0160282 (PMC4976941; doi:10.1371/journal.pone.0160282)
Supplement: S1 Table — (PDF) [file pone.0160282.s006.pdf]

Antigen	Dilution	Manufacturer and catalogue number
Primary Antibody		
β-catenin	1:100	Abcam (ab32572)
Collagen I	1:100	Acris Antibodies (BP8002S)
Collagen IV	1:100	Abcam (ab6586)
CD31	1:6000	DAKO Cytomation (M0823)
Fibronectin	1:100	Abcam (ab23750)
E-cadherin	1:100	BD Transduction Laboratories (610181)
Elastin	1:100	Abcam (ab21610)
Glut1	1:100	Abcam (ab40084)
Ki67	1:100	Abcam (ab16667)
Mucin1	1:100	Imgenex (IMG-80045)
Pan Cytokeratin	1:100	Sigma-Aldrich (C 2562-.2ML)
Vimentin	1:100	Abcam (ab92547)
Secondary Antibodies		
AlexaFluor555 dkαmouse	1:400	Invitrogen (A-31570)
AlexaFluor647 dkαrabbit	1:400	Invitrogen (A-31573)
