# Supplementary material for: Human Organotypic Lung Tumor Models: Suitable For Preclinical 18F-FDG PET-Imaging
Source: PLoS One. 2016 Aug 8;11(8):e0160282. doi: 10.1371/journal.pone.0160282 (PMC4976941; doi:10.1371/journal.pone.0160282)
Supplement: S2 Table — (PDF) [file pone.0160282.s007.pdf]

Specific tissue piece	Method of analysis
Part 1 top middle	*dsDNA, Collagen, Elastin measurement*
Part 1 bottom distal	*dsDNA, Collagen, Elastin measurement*
Part 1 bottom middle	*Histology*
Part 2 top distal	*dsDNA, Collagen, Elastin measurement*
Part 2 bottom proximal	*dsDNA, Collagen, Elastin measurement*
Part 2 bottom distal	*Histology*
Part 3 left	*dsDNA, Collagen, Elastin measurement*
Part 3 right	Histology
Part 3 middle	*Ultrastructure (SEM,TEM)*
